# Dynamic Expression Profiles from Static Cytometry Data: Component Fitting and Conversion to Relative, “Same Scale” Values

**DOI:** 10.1371/journal.pone.0038275

**Published:** 2012-07-12

**Authors:** Jayant Avva, Michael C. Weis, R. Michael Sramkoski, Sree N. Sreenath, James W. Jacobberger

**Affiliations:** 1 Department of Electrical Engineering and Computer Sciences, Case Western Reserve University, Cleveland, Ohio, United States of America; 2 Case Comprehensive Cancer Center, Case Western Reserve University, Cleveland, Ohio, United States of America; Virginia Tech, United States of America

## Abstract

**Background:**

Cytometry of asynchronous proliferating cell populations produces data with an extractable time-based feature embedded in the frequency of clustered, correlated events. Here, we present a specific case of general methodology for calculating dynamic expression profiles of epitopes that oscillate during the cell cycle and conversion of these values to the same scale.

**Methods:**

Samples of K562 cells from one population were labeled by direct and indirect antibody methods for cyclins A2 and B1 and phospho-S10-histone H3. The same indirect antibody was used for both cyclins. Directly stained samples were counter-stained with 4′6-diamidino-2-phenylindole and indirectly stained samples with propidium to label DNA. The S phase cyclin expressions from indirect assays were used to scale the expression of the cyclins of the multi-variate direct assay. Boolean gating and two dimensional, sequential regions set on bivariate displays of the directly conjugated sample data were used to untangle and isolate unique, unambiguous expression values of the cyclins along the four-dimensional data path through the cell cycle. The median values of cyclins A2 and B1 from each region were correlated with the frequency of events within each region.

**Results:**

The sequential runs of data were plotted as continuous multi-line linear equations of the form y  =  [(y_i+1_−y_i_)/(x_i+1_−x_i_)]x + y_i_−[(y_i+1_−y_i_)/(x_i+1_−x_i_)]x_i_ (line between points (x_i_,y_i_) and (x_i+1_, y_i+1_)) to capture the dynamic expression profile of the two cyclins.

**Conclusions:**

This specific approach demonstrates the general methodology and provides a rule set from which the cell cycle expression of any other epitopes could be measured and calculated. These expression profiles are the “state variable” outputs, useful for calibrating mathematical cell cycle models.

## Introduction

The complexity of the cell cycle is apparent to anyone attempting to teach it, describe it, or model it. From one point of view, the cycle is a series of ordered chemical reactions, regulated by feedback and feedforward control systems that are also chemical reactions. For most investigators, the control system is the interesting part of the cell cycle. The number of chemical reactions involved is very large and due to the enzymatic and spatiotemporal nature of these reactions, the complexity is vastly larger. This level of information requires databases and informatics, and the complexity of the network of reaction pathways suggests the need for mathematical models to enable or facilitate system-wide understanding of cell cycle regulation. Models based on systems of ordinary differential equations (ODE) have been developed previously and provide a foundation for larger, more accurate models, e.g., [1], [2].

Measurement of the relative expression of cell cycle regulated epitopes in asynchronous cell populations by cytometry produces data from which relative expression over relative time can be extracted [3]. The general value of this is that, given the appropriate set of markers, the shape or profile of expression over the cycle for any epitope can be evaluated within the context of any others. Often the timing of expression and the shape of the expression profile say something about the period in which a specific epitope is important and/or is a measure of the activities that act on that epitope (proteases, kinases/phosphatases, methylases/de-methylases, etc.). In general, most versions of cell cycle expression profiles are cartoons based on synchronization and bulk measurement methods, e.g., [4], [5]. Since the shapes of these relative expression profiles are equivalent to the outputs of state variables in mathematical models of the cell cycle, they could be used to calibrate and validate mathematical models, if they closely reflected reality - i.e., if they were based on quantitative measurements. In the best case, mathematical models should be calibrated in molecular units, and if not that, then relative units on the same scale. The relative expression of parameters determined from multi-color immunofluorescence cytometry assays, while correlated, are not quantitatively related to each other, except through a tortured path that is difficult to resolve (taking into account fluorophore to antibody ratios, fluorescence quantum yields, photomultiplier spectral responses, fractions of light captured, and run-time instrument settings). Here we present a method to convert multi-color (multi-variate) data to the same relative scale. This is a step toward the goal of molecular scales. We have previously published procedures for converting data for one epitope, measured by cytometry, to molecular scales [6], [7]. If one of the epitopes in a multi-color assay can be converted to a molecular scale, then the procedure described herein will work to convert all of the epitopes in the assay to molecular scales.

The idea here is to measure more than one epitope with indirect assays using the same secondary antibody and using cells sampled from the same experimental pot in each determination. By selecting a cell region in multi-variate data space in which a significant range of expression occurs for each epitope and correlating the assays through an additional measurement, the relative quantities of each epitope can be put on the same relative scale. For the work presented here, we calculated this scale for cyclins A2 and B1, using S phase as the region in which the two cyclins span ranges of expression large enough to be useful, and we used DNA content as the correlating variable. We then calculated the relative expression profiles for cyclins A2 and B1 in a multi-color assay of the same cells in which we measured cyclins A2, cyclin B1, phospho-S10-histone H3 (pHH3), and DNA content. The expression profiles for the cyclins were then converted to the same relative scale. The results show that the two cyclins are expressed at peak at about the same levels. The caveats here are (1) we assume the difference between recognition of the cyclin A2 and cyclin B1 monoclonal antibodies by the secondary polyclonal antibody is negligible; (2) we assume that the differences in affinity for each epitope by the two monoclonal antibodies are negligible, and (3) we assume that epitope exposures are approximately the same - i.e., they are not masked in a biased manner. Given these caveats, this approach is inexact, but likely to not be far off, and in absence of other relatively good approaches, this is a first step.

## Results and Discussion

K562 cells from an exponentially growing culture were fixed then stored in single sample aliquots. One sample was stained with directly conjugated antibodies for cyclins A2 (cycA-PE) and B1 (cycB-A647), phospho-S10-histone H3 (PHH3) and DAPI (4′,6-diamidino-2-phenylindole). Two other samples were stained indirectly for either cyclin A2 or B1 with the same cyclin antibodies (unconjugated), the same anti-mouse Alexa Fluor 488-conjugated secondary, and propidium iodide (PI). We term the sample stained with direct conjugates of cyclin antibodies as “multi-color” and the samples stained indirectly as “single color” samples.

### Data Pre-processing

For multi-color samples, we corrected for spectral overlap between Alexa Fluor 488 (A488) and R-Phycoerythrin (PE) and subtracted background fluorescence using G1 phase immunofluorescence and light scatter (cyclin A2 and cyclin B1 are not expressed in early G1 phase). The latter transformation has the effect of setting the fluorescence of negative cells close to zero. Using DNA content pulse-height and integral, we eliminated events that represent sub-cellular debris and aggregates. Finally, we limited analysis to cells of the 2C stemline by using a gate on a bivariate plot of cyclin A2 vs DNA content of interphase cells. These preprocessing procedures have been previously described in detail [3].

The next section demonstrates segmentation of the continuum of multi-color data that represent cells at all stages of the cell cycle, and thus, the segments or regions are ordered contiguously in one direction from G1 through to cytokinesis. We used pHH3 expression to separate interphase from mitosis, then a bivariate of DNA content vs. cyclin A2 to separate G1, S, and G2 cells. “Movement” through S phase was accomplished by setting contiguous regions on the same bivariate plot. G1 and G2, identified in this manner do not contain bivariate information, but rather point to single parameter histograms for cyclin A2 and cyclin B1. Determination of expression from these histograms was performed by multi-Gaussian modeling, essentially as previously described [8]. There are alternative methods to segmenting these data in which multi-Gaussian modeling is unnecessary [3].

### Extraction of Expression Profiles from Multi-color Data

#### Isolation of mitotic and interphase cell data

**Figure 1:**
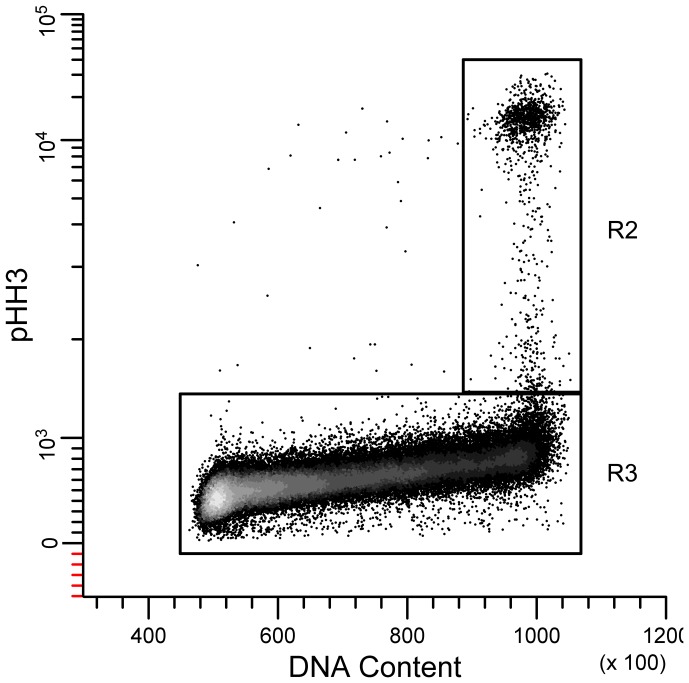
Gating Interphase and mitotic cells. Region R2 defines mitosis and R3 is the first interphase gate.

We divided the cell cycle into interphase and mitotic events by gating bivariate plots of pHH3 versus DNA. We classified as interphase the cells with pHH3 that cluster together from a low value in G1 (2C DNA content) to approximately twice that value in G2 (R3, Figure 1). Cells with higher pHH3 intensity and 4C DNA content were classified as mitotic cells (R2, Figure 1).

#### Segmentation of interphase

**Figure 2:**
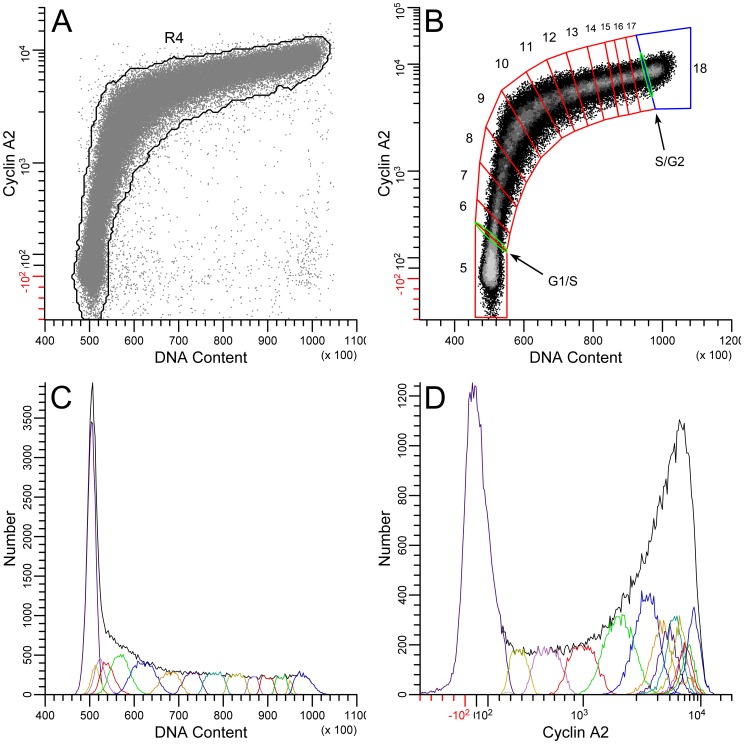
Gating and segmenting interphase. Data were first gated on single cells cells from 2C to 4C in DNA content (not shown) AND (Boolean) R3 (**Figure 1**). (**A**) R4 creates the final gate for interphase. The purpose is to eliminate endoreduplicated or binucleate G1 cells, cyclin A2-negative S phase cells, and outliers. (**B**) Region 5 has been set on G1 cells. S phase has been segmented into regions 5–17, which follow the two dimensional modal backbone of the data. The adjacent boundaries of each region were originally set to be orthogonal to the local slope of the data and then adjusted to create Gaussian character, observed in single parameter histograms of DNA content (**C**) and cyclin A2 (**D**). G2 cells are bounded by region 18 (blue), and thin, oval regions (G1/S, S/G2) were set on the boundaries between G1 and S and S and G2 (green).

The objective here was to assign data in a statistically significant, non-redundant manner with respect to an average cell traversing the data space. There are two aspects to the problem. The first is to isolate in a bivariate plot a track of data that spans an expression range through which cells can be thought to move. The rule is that an individual cell, if it could be tracked, cannot enter a region twice within one cell cycle. Another view is that the data constitute a uni-directional vector with variation about that vector. The second aspect is to determine the two-dimensional variation that defines a unit of information. The approach for the first aspect is to filter the data through a gate as described in the previous paragraph. Thus, the gate, R2, on a bivariate display of PHH3 vs. DNA content isolates interphase cells (**Figure 1**), and the gate, R4 (**Figure 2A**), on a bivariate plot of cyclin A2 vs. DNA content isolates the 2C stem line interphase cells (eliminates 4C, cyclin A2 negative, G1 cells). This last procedure also eliminated outliers (e.g., S phase cells that are negative for cyclin A2). The division of interphase into significant segments is shown in **Figure 2B**. The average cell “moves” from the lower left to the upper right through successive regions starting with region 5, which is equivalent to G1 cells, through to region 18, which is equivalent to G2 cells. The G1 cluster (region 5), identifiable as a two dimensional, approximately normal Gaussian distribution, represents background staining [9]–[11]. The G2 cluster (region 18), unlike the G1 cluster (composed of cells without cyclin A2 expression), represents the distribution of cyclin A2 expression at high levels. The regions 6–17 have adjacent boundaries that started orthogonal to the immediate slope of the bivariate data but were adjusted to obtain as much Gaussian character in both cyclin A2 and DNA content single parameter distributions as possible (**Figure 2C, 2D**). The regions that segment S phase (6–17), in which both DNA and cyclin A2 increase in value, are arbitrary in number with the only constraint being to include a statistically effective number of events. This is not true for the regions that enclose G1 and G2 in which the values of DNA and cyclin A2 either represent measurement variation alone (region 6) or the values of only one parameter that had significant biological variation (region 18). In the case of G1 (region 6), the value of cyclin A2 is approximately zero, and DNA is also a unit value equal to the DNA content of the 2C genome – therefore, the two dimensional distribution is statistical. In the case of G2 (region 18), the two dimensional distribution is statistical for DNA but contains real biological variation for cyclin A2.

#### Extraction of G2 interphase cyclin A2 expression

**Figure 3:**
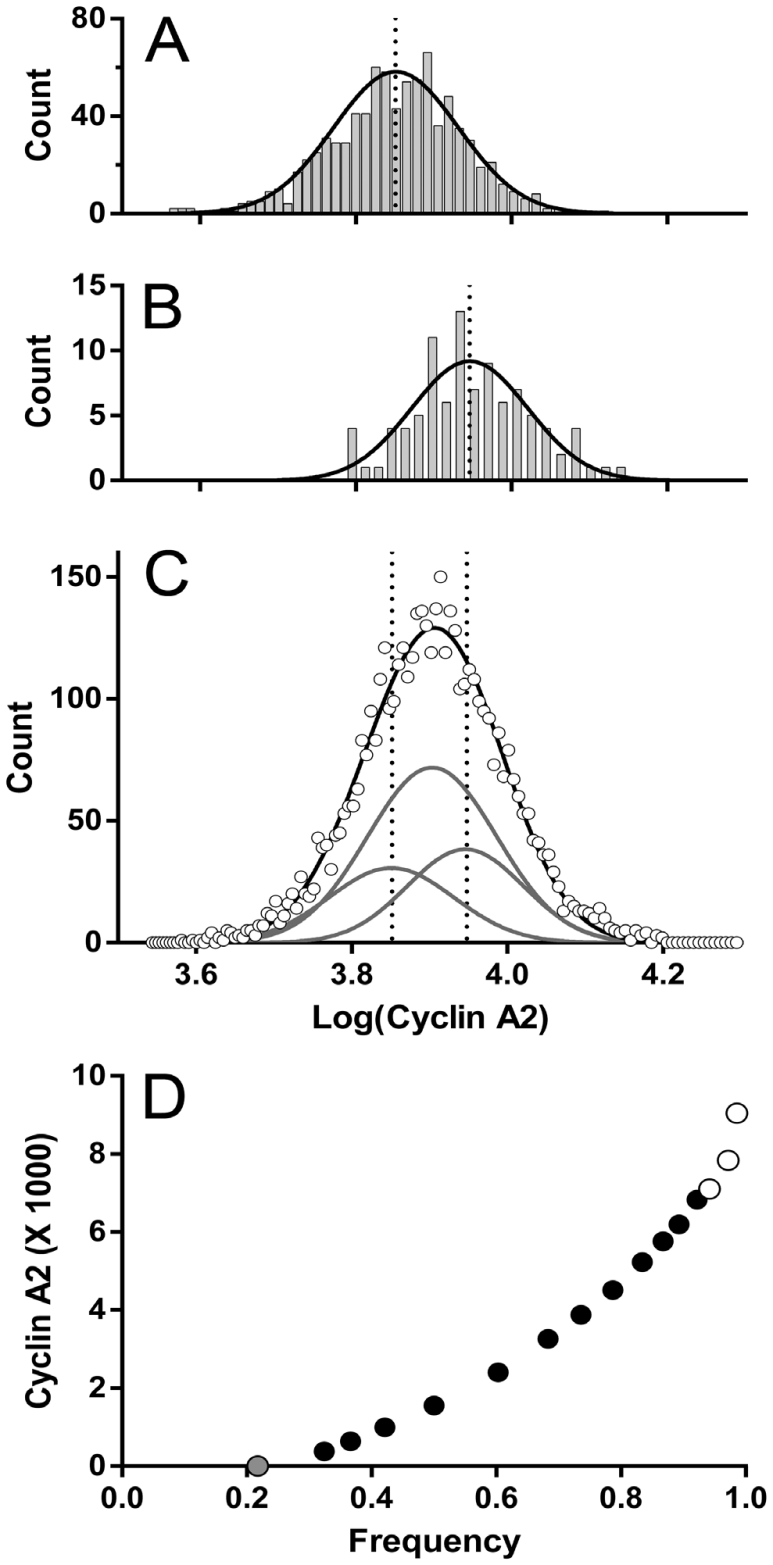
Modeling G2 cyclin A2 expression. The G2 entry (**A**) and exit (**B**) cyclin A2 distributions were gated from the G2/S region (Figure 2B) and G2/M region (Figure 4A), respectively, and displayed as log values. Fitting Gaussian functions to each provided the means and deviations for the multi-Gaussian fit for the G2 distribution (**C**) open symbols  =  data, gray lines  =  component Gaussian functions, and black line is the sum of the component functions). The center function mean was constrained to the middle between the entry and exit means. The deviation was allowed to vary. The amplitudes in the multi-Gaussian model were constrained such that the position of the three points within G2 followed the expression line (**D**) composed of cyclin A2 values derive from the S phase regions in **Figure 2B**. The amplitude constraints allowed variation as needed for fitting. For the fit in C, R^2^ = 97%. The data presented in **D** represents the expression of cyclin A2 in interphase. The values for G1 (gray symbol) was obtained directly from region 5 (**Figure 2B**); the values for S phase (black symbols) were obtained from regions 6–17 (**Figure 2B**), and the values for G2 (open symbols) were obtained as shown in this Figure A, B, and C.

**Figure 4:**
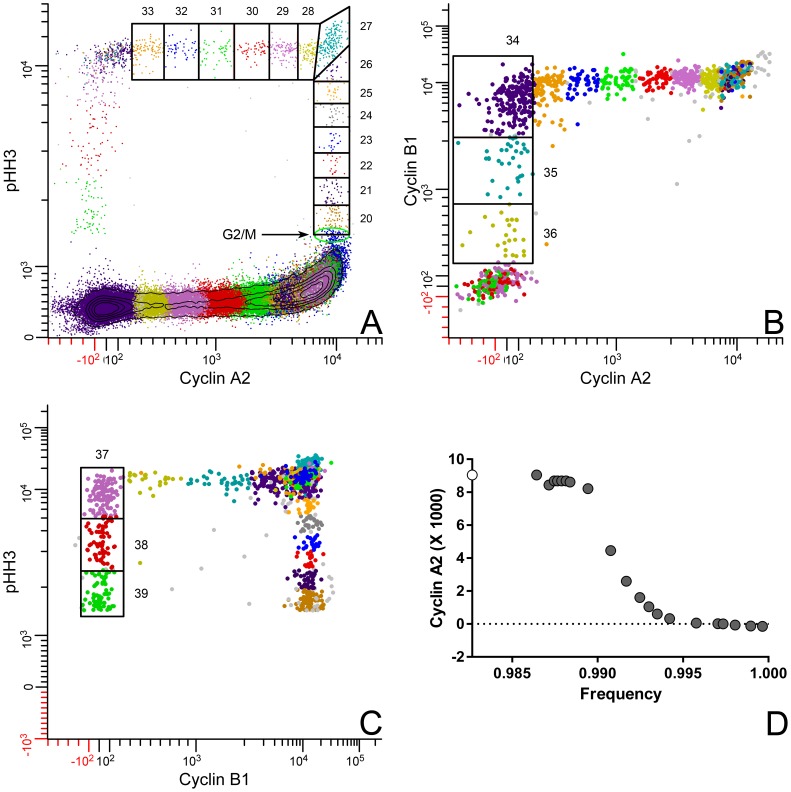
Gating and segmenting mitosis. To segment mitotic cells stained for cyclins A2, B1, and PHH3, it is necessary to view the data in three different bivariate plots. (**A**) mitosis begins with a sharp rise in PHH3. Region 20 marks the start of mitosis. Successive regions can follow the rise in PHH3 to peak levels and the subsequent degradation of cyclin A2. After region 33, cell classification becomes ambiguous, viewed through plots of cyclin A2 vs. PHH3. Color coding of data is arbitrary with colors assigned by combinations of region and gate values. Clusters with ambiguous assignment display as multi-colored clusters. (**B**) The ambiguity in (**A**) is resolved by a plot of cyclin A2 vs. B1, gated on R2 (**Figure 1**). Regions 34–36 track the degradation of cyclin B1 and end at the next ambiguous cluster. (**C**) The last ambiguous cluster is resolved with a plot of PHH3 vs. cyclin B1. The rules for drawing regions are described in the text and in the legend of **Figure 2**. The expression of cyclin A2 through mitosis was calculated, either directly or indirectly from the mitotic regions, 20–36, as described in the text and plotted in (**D**). The white symbol represents the last G2 value (**Figure 3**). Gray symbols represent mitotic values.

To calculate the change in cyclin A2 over the span of the G2 cyclin A2 distribution, we fit the sum of multiple Gaussian functions to the G2 distribution. This prevents inclusion of statistical error as expression values. The distributions of cyclin A2 at the entry and exit “points” of G2 were the basis for the Gaussian component variables (coefficient of variation (CV)). These “points” (regions) are illustrated in **Figure 2B** (green oval region  =  G2 entry) and **Figure 4A** (green oval  =  G2 exit). The corresponding cyclin A2 distributions are plotted in **Figure 3A and 3B**. The G2 cyclin A2 distribution is plotted in **Figure 3C** with the multiple Gaussian fit. The entry and exit points are denoted by dotted lines. To find the signal in the G2 phase, a constrained optimization problem was framed to minimize the mean squared error between the data histogram of cyclin A2 (**Figure 3C**) and the sum of multiple, weighted Gaussian components. The means and standard deviations of the “entry” and “exit” components were calculated by fitting Gaussian functions to the cyclin A2 distributions from the S/G2 and G2/M regions (**Figure 3A and 3B** **)**. The center component was constrained to the center of the distance between the entry and exit points. The standard deviation was allowed to vary. The integrals of each component were constrained to achieve an approximate continuity of the expression line plotted in **Figure 3D**. Here, the reasoning is the idea behind Occam’s razor. It should be noted that rates of cyclin accumulation in K562 G2 cells are less than is observed in other common cell lines (e.g., Molt4), and that here, the G2 cyclin A2 or B1 distributions have a unimodal Gaussian shape (without distinct shape features like multiple modes or skewness), and thus have several solutions as a series of three Gaussian sub-components with variable integral values. Therefore, the guide we used was the expected extrapolation of the trajectory of expression from S phase (**Figure 3D**). Finally, the expression profile for interphase was calculated and plotted as in **Figure 3D**. The G1 and S values were calculated directly from the regions 5–17 in **Figure 2A**. The G2 values were the means of the Gaussian fitting sub-components.

#### Segmentation of M

Mitotic cells were analyzed by contiguous regions in a similar but more complex manner as that for interphase S cells. In this case, contiguous regions cross bivariate views of the data (**Figure 4A–C**). Starting with region 20 (**Figure 4A**), the regions follow the rise in PHH3 as cells enter mitosis, pass through the state of maximum PHH3 and cyclin A2 (region 27), and then follow the cells that were actively degrading cyclin A2 at the time of fixation (regions 28–33). At that point, we reach an ambiguous state from the cyclin B1 point of view. Therefore, we switch to a bivariate plot (**Figure 4B**) of cyclin B1 vs. cyclin A2 gated on R2 (**Figure 1**) to follow the cells that were actively degrading cyclin B1 (regions 34–36). Subsequent to region 36, we again reach a state of ambiguity from a PHH3 point of view. Segmentation is resumed again on a bivariate plot of PHH3 vs. cyclin B1 (**Figure 4C**), and regions 37–39 complete the trajectory through mitosis. If gates in three dimensions (PHH3, cyclin A2, and cyclin B1) could be drawn, these gates would be contiguous within one 3D histogram. The bounding and transition regions (27, 34, 37, and 39) are equivalent problems to the G1 and G2 regions (5, 18) for interphase. Both of these regions need to account for statistical variation rather than significant biological change. Given the shape of the cluster within region 27, it is likely that the programmed expression of both cyclin A2 and PHH3 increase and then decrease, but this is a guess and without further support this cluster represents one point in time for both parameters. To pursue the idea, we need another marker that would separate the rise and fall in the same manner that cyclin A2 separates the rise and fall of PHH3.

For the preceding statements, there is abundant support in the literature. First, phosphorylation of histone H3 at serine 10 increases dramatically either at the end of G2 or the beginning of mitosis [12]. This is likely a function of aurora kinase B [13], [14]. Second, the degradation of cyclin A2 occurs after nuclear membrane breakdown, mediated by Cdc20 and the anaphase promoting complex/cyclosome (APC/C). This process produces data that vary for cyclin A2 but not B1. In a third step, cyclin B1 is degraded through a similar process, initiated by Cdc20 and enforced by Cdh1. Finally, histone H3 is incompletely dephosphorylated by phosphatases, and cells divide with residual phosphorylation [15]. For more detail on this complex, and as yet incompletely understood process, see [16]–[18]. At the end of M (region 39), both cyclins are below the threshold of detection, and PHH3 is also about 2× it’s initial G1 level.

Although the M phase data for cyclins A2 and B1 create an orthogonal pattern shown in **Figure 4B**, this is not always the case. If the onset of cyclin B1 degradation occurs prior to the complete loss of cyclin A2, then a more curved relationship will be observed. In that case, region setting would be as shown for S phase (**Figure 2B**) - i.e., the regions would follow the local bivariate slope.

#### Extraction of mitotic cyclin A2 expression

After segmenting M, expression of cyclin A2 is read directly from regions 20–36 and indirectly from regions 37–39. Indirect reading of parameter values tacitly assumes that the relationship between the exposed and hidden parameters is orthogonal. In this case, that assumption is true. However, we have found that the error involved in making this assumption when orthogonality is not true is acceptable. This is likely because the center values that are derived are not heavily dependent on the exact shape of the region. An alternative is that the relationships are not sufficiently deviated from orthogonality to make a large difference. The expression of cyclin A2 in mitosis is shown in **Figure 4D**.

#### Extraction of cyclin B1 expression

**Figure 5:**
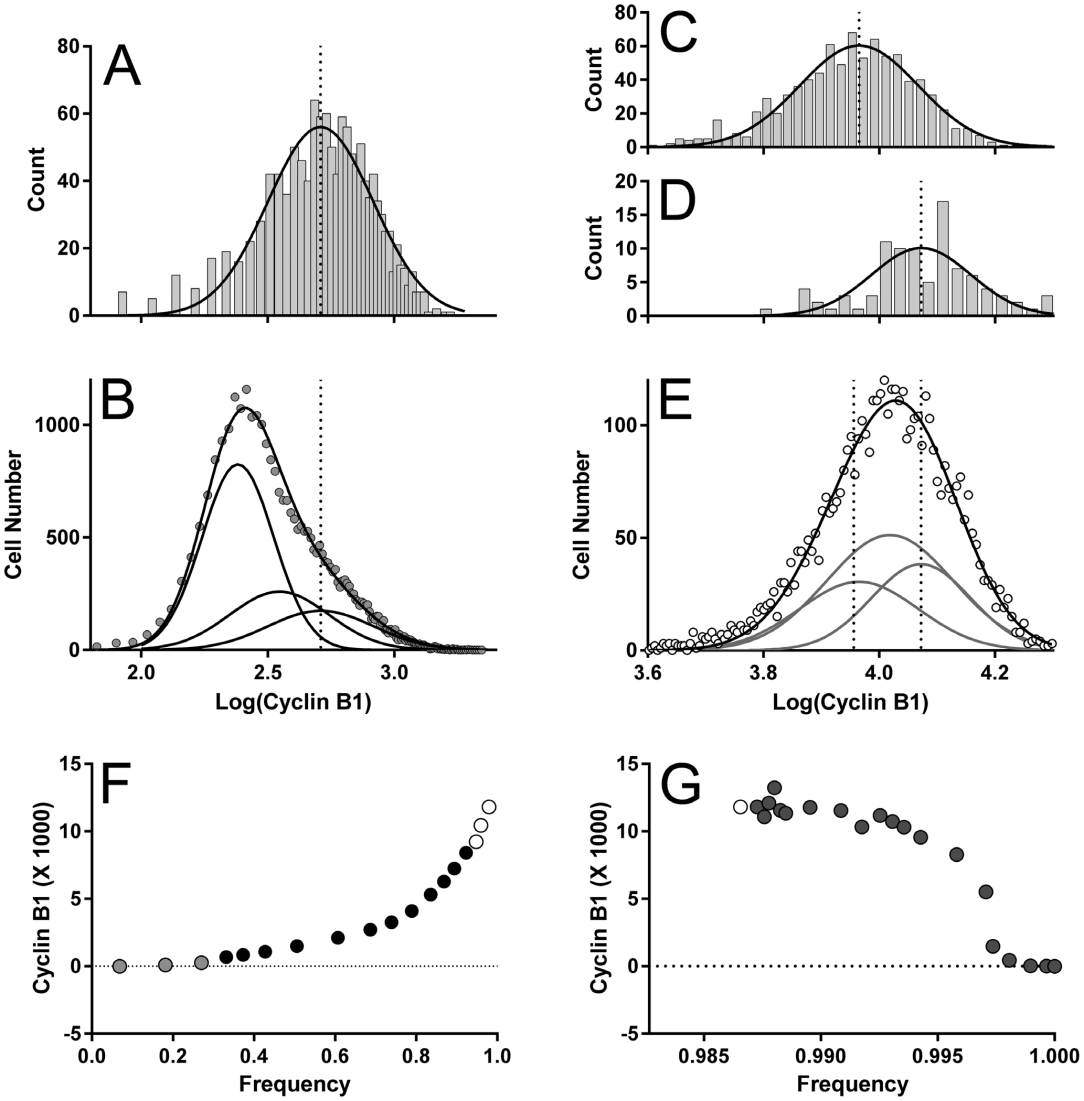
Modeling cyclin B1 expression in G1 and G2. This figure is analogous to **Figure 3**. (**A**) The distribution of cyclin B1 expression at the exit point for G1 was gated from the G1/S region (**Figure 2B**), converted to log values, and fit with a Gaussian function to determine mean and deviation. (**B**) The values obtained from the fit in (A) were used to constrain the position and width of the exit position in the distribution of cyclin B1 fluorescence of G1 (gated from region 5, **Figure 2B**). The peak position of this distribution was set as the lower (background) level position, using the CV of the exit function. A center component was constrained with the same CV and a middle position between the lower and exit components. Amplitudes were loosely constrained to achieve continuity within the cyclin expression curve, as in **Figure 2**. (**C**, **D**) The regions, S/G2 and G2/M, (**Figure 2B**, **Figure 4**) were used to gate the cyclin B1 distributions for entry to and exit from G2. Each distribution was fit to a Gaussian function. (**E**) The means and deviations of these functions were used to constrain the lower and upper Gaussian components of a three part function to fit the G2 distribution of cyclin B1. The same constraints on amplitude were applied as for the cyclin A2 distribution. (**F**) Interphase and (**G**) mitosis expression plots for cyclin B1 were determined as in **Figure 2** for interphase and as in **Figure 4** for mitosis. Gray symbols  =  G1 determinations. Black symbols  =  S phase values. White symbols – mitosis determinations. G1 and G2 values were from Gaussian fitting as shown in B and E.

**Figure 5** illustrates the same logic for extracting the Cyclin B1 expression information as shown in **Figure 3** for cyclin A2. In this case, since cyclin B1 is expressed in late G1 [19], [20], both G1 and G2 require deconvolution. **Figure 5A** shows the G1 exit cyclin B1 distribution gated from the bivariate plot of cyclin A2 vs. DNA and region, G/S (**Figure 2B**), and the Gaussian function fit to the data. **Figure 5B** shows the G1 distribution, gated similarly from region 5 (**Figure 2B**). In this case, we do not have entry and exit information, but there is sufficient shape information to allow a constrained Gaussian at the G1 exit point, a lower Gaussian fit to the peak, and a centered function between the lower and upper means. **Figure 5C and 5D** shows entry and exit distributions gated from the border regions (S/G2, G2/M) shown in **Figures 2B** and **4A**. Fitting the regions to the G2 distribution, gated from region 18 (**Figure 2B**) is shown in **Figure 5E**. The same fitting problems and solutions as were encountered in fitting Gaussian functions to the G2 distribution of cyclin A2 apply here. Additionally, since we gated interphase from bivariate plots of parameters other than cyclin B1, the orthogonality assumption (discussed earlier) applies. Another solution would have been to independently set regions on a bivariate of cyclin B1 vs. DNA. Using current software, the benefit in accuracy does not outweigh the cost in trying to obtain a one-to-one match for the G1 and G2 regions (5 and 18 in **Figure 2B**). Extraction of cyclin B1 information by both direct and indirect methods was performed as for cyclin A2. Interphase expression is shown in **Figure 5F** and mitosis expression in **Figure 5G**.

### Same Scale Correction

#### Correction method

To put each cyclin on the same relative scale, we used identical samples of K562 cells indirectly stained for each cyclin using the same secondary antibody. Both primary cyclin antibodies had been previously titered and were used at optimal (slightly subsaturated) concentrations. Both antibodies saturate within a 2 fold dilution of each other and bound antibodies have very long half-lives (6 and 15 days for GNS1 and 11B2G3, respectively). See the Introduction for a brief discussion of the logic and assumptions made here.

**Figure 6:**
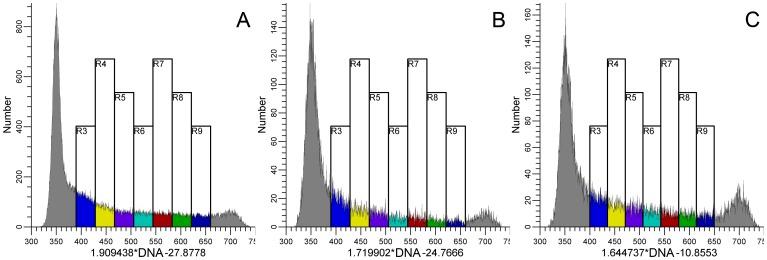
Equivalent regions set through S phase. DNA histograms from K562 cells stained for single cyclins A2 (B) or B1 (C) or stained for both cyclin A2, B1, and PHH3 (A) were transformed by functions denoted on the X axes to place the mode of G1 in channel 350 and the mode of G2 in channel 700. Each histogram was then segmented with 6 equally sized regions that expanded out from a middle point. The median values for cyclin A2 and B1 were calculated from measurements gated on these regions. These values were then used to construct the plots in **Figure 7A, 7B**.

To put the correlated multi-color data on a single color scale, we used single parameter, DNA content histograms for both single color experiments and the multi-color data to map the cyclin data in the same relative data space in each file. The high precision with which DNA content can be measured allowed us to map the same data regions in three separate files with confidence. To do this, we determined the G1 and G2 modes, then using linear regression, we transformed the DNA data in each file to stretch from modal values of 350 to 700. That allowed us to create a series of seven gates, centered in S phase and spanning the S phase rise in each cyclin. The process is illustrated in **Figure 6**.

**Figure 7:**
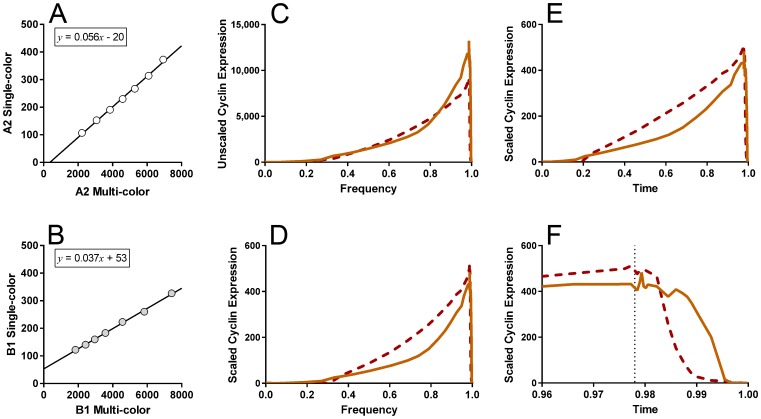
Expression profiles of cyclins on the same scale. The data presented piece-wise in **Figures 3D**, **4D**, **5F, and 5G** were transformed to set the first and last values (frequency = 0 and 1) to zero in accord with the known low levels (below detection) of these cyclins at the end and beginning of the cell cycle (**C**). The functions in (**A**) and (**B**) were then used to transform the data in (**C**) to the form in (**D**), which set them to the same relative scale, and therefore, the amplitudes now have meaning. In (**E**) and (**F**) the frequency data were transformed from frequency to time as explained in the text. Dotted line in (F) denotes the boundary between G2 and M.

The correlations for each cyclin in multi- and single color modes are shown in **Figure 7A and 7B**. Scaling the cyclin data changes the meaningless amplitudes (**Figure 7C**) to values that are estimates of the relative ratios between cyclins A2 and B1 through the cell cycle (**Figure 7D**). In this case, putting the cyclins on the same scale shows that they are expressed at ∼equivalent peak levels.

#### Cell cycle expression of cyclins A2 and B1

Each of the defined regions or model components in the preceding analyses has an associated frequency of events that is proportional to the time that the nominal or average cell spends in that state [21]–[23], with state defined by the levels of expression of DNA, pHH3, cyclin A2, and cyclin B1. To create expression profiles, we calculated the region/component associated cumulative frequency for each as previously described [3], [7]. A plot of the frequencies and associated same-scale expression levels of cyclins A2 and B1 is shown in **Figure 7D**. To convert frequency to relative cell cycle time, we have to correct the frequency values by the population age distribution, which in the perfect case follows an exponential decline from a factor of 2 to 1 from the beginning of the cycle (t = 0) to the end of the cycle (t = 1) in units of cell cycle time. The reason for this is that 1 mitotic cell gives rise to 2 G1 cells. In **Figures 7E and 7F**, the cyclin expression data are plotted transformed to a time scale. These figures show that the two cyclins peak together in late G2 and that cyclin A2 decreases prior to cyclin B1 (**Figure 7F**).

#### Final heuristics

Since we calculate the center frequency and expression values for each region/component, the boundaries at transitions along the expression profile can be improved. For example, for cyclin A2 measurement in G1, we calculate only one central value. However at frequency or time equal to zero (f = 0. t = 0), the same value holds. Therefore, we improve the profile by adding that value at f = 0, t = 0. Equally to improve the profile at the G1/S boundary for cyclin A2, we have extrapolated from the first three values in S to the X frequency or time point at Y = 0 and placed a value there. Since we are not certain of the shape of cyclin A2 at the S/G2 boundary, we do not try to improve that transition point. However, we can use the same logic to improve the S/G2 boundary for DNA content. The occasions when these improvements add value are points where there is a large number of events within a region, and therefore, the expression data are sparse.

#### Expression of cyclins A2 and B1 in context

**Figure 8:**
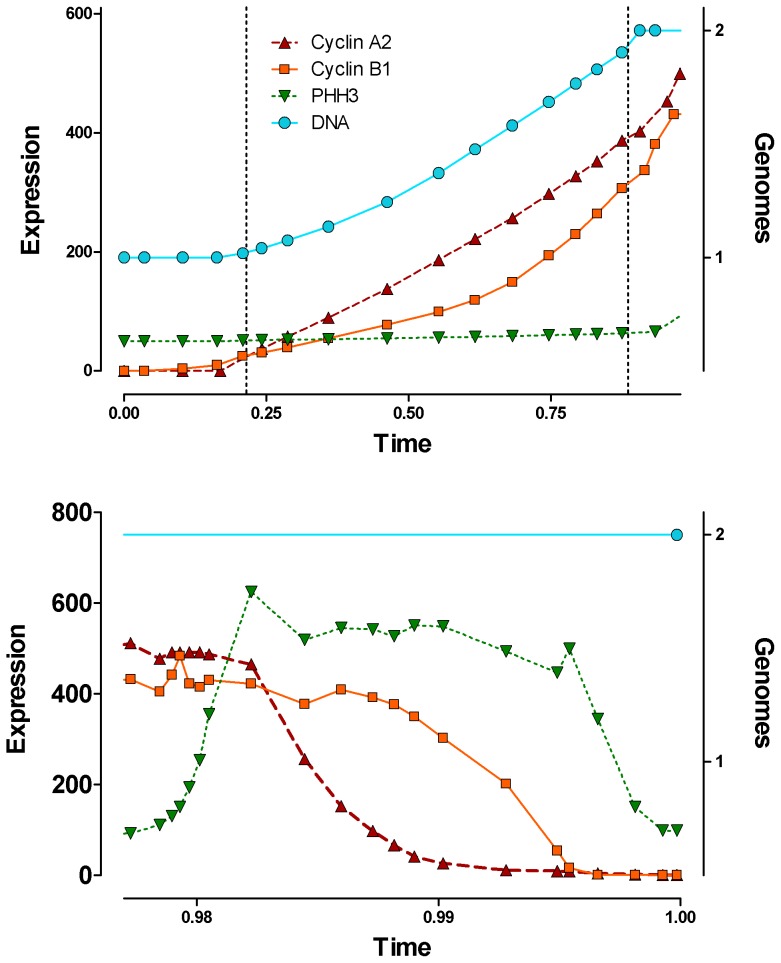
Cyclin expression in context. The cyclin data from **Figure 7E, 7F**, are co-plotted with PHH3 and DNA expression. The cyclins are on the same relative scale. DNA is on an absolute scale (Genomes) and PHH3 was transformed to an arbitrary relative scale that denotes that interphase levels of PHH3 are not zero. Interphase is shown in (**top panel**), and M is shown in (**bottom panel**). Dotted lines mark the G1/S and S/G2 boundaries as calculated from DNA histograms by conventional DNA analysis using a broadened polynomial to fit S phase.

Figure 8 shows the scaled expression of cyclins A2, B1, DNA content, and PHH3. The cyclins were scaled relative to each other. DNA content was scaled in absolute terms (genomes), and PHH3 was scaled in an arbitrary manner to show that the interphase levels are not zero [24] and the range of mitotic levels relative to that - i.e., no magnitude relationship to the cyclins.

### Final Comments

Here we have demonstrated how to extract cell cycle expression profiles from flow cytometry data. The point to this exercise is the generation of dynamic expression data from unperturbed, asynchronous cell populations to support mathematical modeling of the cell cycle [25]. The models that we have considered first, and those to which this work clearly relates, are ODE models, e.g., [1], [2]. We have recently examined the literature for such models and identified 154 models [25] with a large subset listed in [26]. Most models focus on parts of the cell cycle (e.g., G1 transit), but a small subset model complete cell cycle transition [1], [2], [27]. These are the models that would most clearly benefit from availability of profiles such as those presented here. While we consider the ability to extract quantitative expression profiles a step in the right direction, it is clear that modeling the molecular reaction network that regulates the cell cycle needs to include movement of molecules on and off substrates (in and out of compartments). There are many examples of molecules translocating upon activation, but even more demanding in terms of understanding is the use of proteins, especially enzymes, that migrate and change roles as a function of cell cycle progression. The chromosome passenger complex, of which Aurora kinase B is a part, is one such example of this, e.g., [28]. The ability to integrate the work here with cellular localization can be done, albeit with some development, by using laser scanning cytometry, e.g., [15]. Finally, we would like to mention that it is possible using statistical methods and large expression measurements on a few cells (e.g., RNA expression arrays) to obtain similar expression profiles (Omar De La Cruz Cabrera, unpublished work). This latter approach appears to be a more formal general case of the empirical approach employed herein.

## Materials and Methods

### Cells and Cell Culture

The human hematopoietic cell line, K562 [29], was obtained from Keith Shults and cultured as previously described [30].

### Fixation

Exponentially growing cells were washed with phosphate buffered saline (PBS), resuspended at 2 million cells per 50 ul PBS aliquots and fixed with 450 ul MeOH as previously described [31].

### Antibodies

Anti-phospho-S10-histone H3 rabbit antibodies, conjugated to Alexa Fluor 488 (#9708) was purchased from Cell Signaling Technology, Waverly, MA) and used at manufacturer’s recommended concentration. Anti-cyclin A2 unconjugated and conjugated to R-phycoerythrin (PE) were gifts from Vince Shankey (11B2G3, Beckman Coulter, Miami, FL) and used at 0.125 ug per reaction; anti-cyclin B1 (GNS1) was purchased unconjugated from BD Biosciences and conjugated in the laboratory to Alexa Fluor 647 with a kit from (Molecular Probes/Invitrogen, Carlsbad, CA) and used at 0.0625 (conjugated) or 0.125 ug per reaction (unconjugated). Secondary antibody was affinity purified goat anti-mouse conjugated to Alexa Fluor 488 (Invitrogen, Carlsbad, CA).

### Immunofluorescence Staining

2 million fixed cells were used per reaction. Direct, multi-color staining has been described exactly for these markers [32]. Indirect staining used the same antibodies with the following alterations. Phospho-S10-histone H3 antibody was not used; cyclin A2 or cyclin B1 antibodies were used in separate samples; secondary antibody was used at 2.5 × the primary antibody amount; propidium di-iodide (Calbiochem-Behring, La Jolla, CA) at 25 ug/ml was used to stain DNA after treatment with RNase (Sigma, St. Louis, MO) for indirect assays. In multi-color samples, DAPI was used at 1 ug per sample in 1 ml PBS in multi-color assays (Invitrogen).

### Flow Cytometry

We used a BD Biosciences LSR II instrument with stock filters supplied by the manufacturers and with ultraviolet (355 nm), violet (405 nm), blue (488 nm), and red (633 nm) lasers. PE data was compensated for spectral overlap from Alexa Fluor 488 offline.

### Software

WinList 7.0 (Verity Software House, Topsham, ME) was used as the primary data processing tool. ModFit LT 3.0 was used for DNA analysis. CytoSys [33] running inside of MatLab 8.0 (MathWorks, Natick, MA) or Prism 5.0 (GraphPad Software, La Jolla, CA) were used for multiple Gaussian fitting. Prism was used for linear regression. CytoSys or Prism were used to synthesize expression profiles with a uniform number of interpolated points, but the profiles amount to point to point connections. Microsoft Excel (Redmond, WA) was used for all other calculations.
